# Measuring Virgin Female Aggression in the Female Intruder Test (FIT): Effects of Oxytocin, Estrous Cycle, and Anxiety

**DOI:** 10.1371/journal.pone.0091701

**Published:** 2014-03-10

**Authors:** Trynke R. de Jong, Daniela I. Beiderbeck, Inga D. Neumann

**Affiliations:** Department of Behavioral and Molecular Neurobiology, University of Regensburg, Regensburg, Germany; Tulane University Medical School, United States of America

## Abstract

The costs of violence and aggression in our society have stimulated the scientific search for the predictors and causes of aggression. The majority of studies have focused on males, which are considered to be more aggressive than females in most species. However, rates of offensive behavior in girls and young women are considerable and are currently rising in Western society. The extrapolation of scientific results from males to young, non-maternal females is *a priori* limited, based on the profound sex differences in brain areas and functioning of neurotransmitters involved in aggression. Therefore, we established a paradigm to assess aggressive behavior in young virgin female rats, i.e. the female intruder test (FIT). We found that approximately 40% of un-manipulated adult (10–11 weeks old) female Wistar rats attack an intruder female during the FIT, independent of their estrous phase or that of their intruder. In addition, adolescent (7–8 weeks old) female rats selected for high anxiety behavior (HABs) displayed significantly more aggression than non-selected (NAB) or low-anxiety (LAB) rats. Intracerebroventricular infusion of oxytocin (OXT, 0.1 µg/5 µl) inhibited aggressive behavior in adult NAB and LAB, but not HAB females. Adolescent NAB rats that had been aggressive towards their intruder showed increased pERK immunoreactivity (IR) in the hypothalamic attack area and reduced pERK-IR in OXT neurons in the paraventricular hypothalamic nucleus compared to non-aggressive NAB rats. Taken together, aggressive behavior in young virgin female rats is partly dependent on trait anxiety, and appears to be under considerable OXT control.

## Introduction

The prevalence, negative impact and costs of violence and aggression in our society have stimulated the scientific search for the predictors and causes of aggression, as well as possible treatments to reduce harmful violence. It has long been believed that aggression is mainly a problem among (especially young) males [1]–[3]. However, rates of violence and conduct disorder diagnoses in adolescent girls (aged 12–18 years) and young women (aged 18–24 years) are considerable [4], [5] and have risen in Western society in recent years [6], [7]. In addition to the damage done to the victims, the negative consequences of aggression extend to the perpetrators (both male and female): the display of increased aggressive behavior in childhood and adolescence is associated with substance abuse, lower socio-economic status, various social problems, and impaired physical health in adulthood [8]–[11].

These findings signal the need to increase the understanding of the neurocircuitry underlying aggression in both sexes, for example by making use of animal models of aggressive behavior. Although such translational studies have made great progress in the understanding of inter-male aggression in recent years, experiments focusing on females are lagging behind [12]–[14]. This may be a problem, as results obtained in males are not necessarily representative for females. For example, the main hormones and neurotransmitters involved in inter-male aggression, namely testosterone, serotonin (5-HT), and vasopressin (AVP) [13], [15]–[18], have profound sex-specific effects on social behaviors [19]–[23], including aggressive behavior [24]–[26]. In addition, many brain areas that are implicated in both inter-male and inter-female aggression, including the lateral septum (LS), the bed nucleus of the stria terminalis (BST), the anterior and ventromedial hypothalamus (AHA, VMH), the amygdala and the paraventricular hypothalamic nucleus (PVN) [15], [27]–[31] are sexually dimorphic [32]–[33] and may control aggressive behavior in a sex-dependent manner. Due to the anticipated low levels of aggression in females from gregarious species, the (relatively rare) studies of female aggression have focused on lactating female rodents that show maternal defense [34]–[38], or on females from highly territorial rodent species such as Syrian hamsters (*Mesocricetus auratus*) and California mice (*Peromyscus californicus*) [39]–[41]. Although these studies have provided considerable knowledge on the neurobiology of aggression in lactating or territorial females, the extrapolation to non-maternal females of gregarious species is *a priori* limited: maternal aggression is under strong influence of parturition-associated neuroendocrine adaptations [42] and the neurobiological control of offensive behavior in highly territorial species may differ from that of gregarious, group-living species such as rats and humans [43].

The main aim of the present study was, therefore, to establish and validate a suitable paradigm to measure aggression in adolescent and young adult (7–11 weeks old) virgin female rats, i.e. the female intruder test (FIT). We focused on this age group to match the age at which aggression peaks in girls [44] and at which effective treatment would be most beneficial. We anticipated that only a minority of the female residents would attack an intruder [45], putatively influenced by the estrous phase of residents and/or intruders as well as the level of innate anxiety of the residents. More precisely, we hypothesized that receptive (pro-estrous/estrous) residents would be less aggressive than non-receptive (met-estrous/di-estrous) residents [46] (but see [47]). Furthermore, we hypothesized that female Wistar rats selectively bred for high (HAB) or low (LAB) anxiety-related behavior (as measured in the elevated plus-maze (EPM) and other anxiety tests [48]) would display higher levels of aggression compared to non-selected (NAB) rats, as has been shown in male HAB and LAB rats [49].

In a follow-up experiment, we assessed the sensitivity of the FIT to pharmacological manipulation. Thus, we explored the behavioral effects of intracerebroventricular (ICV) infusion of oxytocin (OXT), a nonapeptide that has been shown to facilitate many social behaviors, both in rodents (social memory [50], social preference [51], pair bonding [52], maternal care and maternal aggression [42], [53]) and in humans (face recognition [54], trust [55], empathy [56] and social reinforcement-induced learning [57]). OXT has been found to reduce aggression in female Syrian hamsters [58] and in male wild type Groningen rats selected for high aggression [59]. In addition, intranasal application of OXT reduced aggressive behavior in adult women with high levels of anxiety, but was ineffective in women with normal levels of anxiety [60].

Finally, we aimed to compare regional activation patterns in adolescent female NAB rats that had behaved either aggressively or non-aggressively toward an intruder in the FIT. Inspired by previous studies in male and female California mice [39], [61], [62], we analyzed the level of phosphorylated extracellular signal-regulated kinases (pERK) immunoreactivity (IR) in various brain areas known to be involved in aggressive behavior.

## Methods

### Ethics Statement

Experiments were approved by the Committee on Animal Health and Care of the Government of the Oberpfalz and are in accordance with the Guide for the Care and Use of Laboratory Animals produced by the National Institute of Health.

### Animals

Experiments were carried out in female Wistar rats, which were bred in the animal facilities of the University of Regensburg, Germany and were either non-selected (NAB), or selectively bred for high (HAB) or low (LAB) anxiety-related behavior [49]. Intruder females were NAB Wistar rats obtained from Charles Rivers Laboratories (Sulzfeld, Germany). All rats were kept under controlled laboratory conditions (12∶12 h light/dark cycle; lights off at noon, 21±1°C, 60±5% humidity, standard rat nutrition (RM/H, Ssniff Spezialdiäten GmbH, Soest, Germany) and water *ad libitum*) and housed in groups of 4–6 in standard rat cages (55×35×20 cm) with sawdust bedding until the start of the experimental procedures.

Male rats were not used in this set of experiments, but data from adult (16–22 week old) male Wistar rats (n = 108) generated in previous experiments were used here for illustrative purposes (see [49] and [63] for details on housing conditions).

### Overview of Experiments (Fig. 1)

In exp. 1, general aggressive behavior and the link between aggression and the estrous cycle were assessed. Young adult NAB females (n = 69) underwent their first FIT at 10–11 weeks of age. The behavior of the females was compared with the behavior in the RI-test of 108 unmanipulated, naïve adult male NAB rats (a subset of the 176 males described in [63] for which attack frequencies were recorded). Vaginal smears were taken from both residents and intruders within one hour after completion of the FIT. This resulted in four experimental groups: receptive residents with receptive intruders (n = 15), receptive residents with non-receptive intruders (n = 20), non-receptive residents with receptive intruders (n = 11) and non-receptive residents with non-receptive intruders (n = 23).

**Figure 1:**
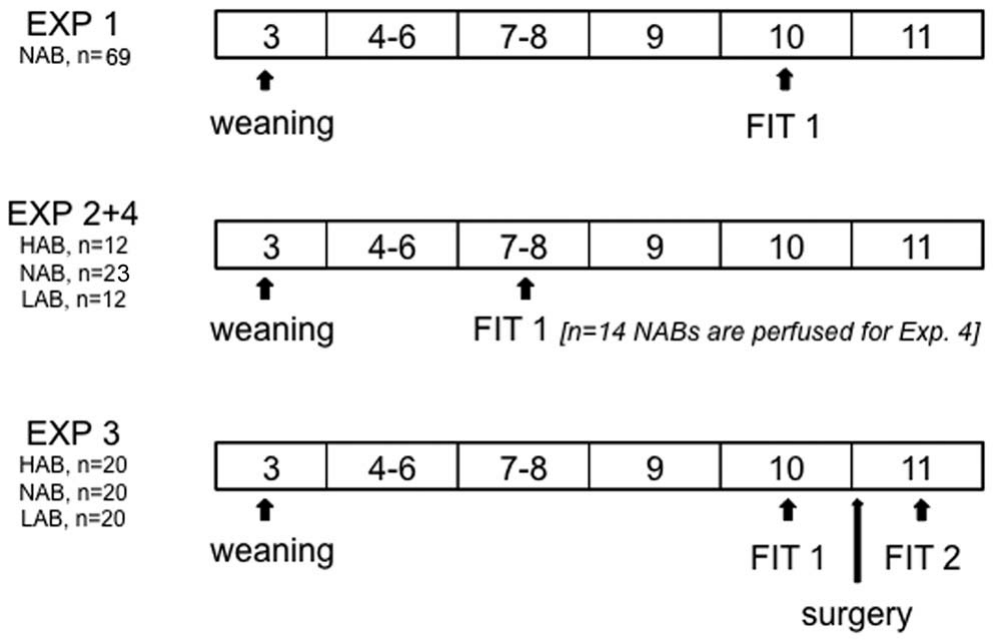
Timeline of experiments.

In exp. 2, the link between aggressive behavior and anxiety was assessed in adolescent (7–8 weeks of age) female NAB (n = 23), HAB (n = 12) and LAB (n = 12) rats. The rats were tested for anxiety behavior on the EPM and, two days later, for aggressive behavior in the FIT.

In exp. 3, adult (10–11 weeks old) female NAB (n = 20), HAB (n = 20) and LAB (n = 20) females were tested in the FIT (“FIT 1”), placed back into their original group cages within 1 hour after FIT 1, and were left alone until surgery. Five days after FIT 1, the females underwent stereotaxic surgery for implantation of the ICV guide cannula and were allowed to recover in single observation cages for two nights. Females were divided into two treatment groups (VEH and OXT) per rat line based on their behavior in FIT 1, to ensure comparable baseline attack frequencies, attack latencies and percentage of aggressive behavior. Twenty min prior to FIT 2, the rats received an ICV infusion with either vehicle (VEH) or synthetic OXT. Group sizes were n = 10 per treatment and rat line.

In exp. 4, immunoreactivity of pERK alone and of pERK in OXT neurons was assessed in non-aggressive (n = 7) and aggressive (n = 7) adolescent female NAB rats – a subset of the rats tested in exp. 2. Females were selected for IHC analysis as follows: non-aggressive females had not attacked their intruder and had displayed aggressive behavior for less than 10% of the time in the FIT; aggressive females had attacked their intruder and had displayed aggressive behavior for more than 20% of the time. Both groups contained approximately equal amounts of receptive/non-receptive residents and intruders.

### Female Intruder Test (FIT)

The Female Intruder Test was adapted from the resident-intruder test (RI-test) in males as performed in our laboratory (see [63] for a detailed description). Similar to RI-tests, the FITs took place in the early dark phase (13.00–16.00 h) under dim red light conditions. Whereas adult male residents are typically housed with a female cage mate in an experimental observation cage (40×24×35 cm, Plexiglas walls) for ten days prior to the RI-test, in the adapted paradigm the experimental females were group-housed with age-matched females since weaning and were isolated in experimental observation cages (same as the males) for two nights (ca. 48 h) prior to the FIT. These females were also termed “residents”, although in contrast to the males we do not know whether they consider the observation cage as their territory. The FIT started when a female intruder (unfamiliar to the test female and 10–30% less body weight – the differences in body weight between residents and intruders were always balanced over experimental groups) was placed into the observation cage. The duration of the test was 10 min, which has been shown to yield reliable information about individual aggression levels in the RI-test [64]. Behavior was videotaped, and ongoing behavior was continuously scored from video by an experienced observer blind to line/treatment using the JWatcher event-recorder program [65]. Similar to RI-tests, we scored the attack frequency and latency to first attack, as well as percentage of time displaying the following behaviors: attack, threat, lateral threat, offensive upright position, keeping down the intruder, and offensive grooming of the intruder (these six behaviors were pooled as “aggressive behavior”), social investigation (olfactory or tactile investigation of the intruder that was not aggressive, defensive, or sexual in nature), defensive behavior (submissive posture, kicking a following intruder with hind limb), sexual behavior (mounting, hopping, darting, lordosis), cage exploration, autogrooming, eating/drinking, and immobility (these last four behaviors were pooled as “non-soclal behavior”). Autogrooming was also separately analyzed as a potential marker of anxiety or OXT-induced stereotyped behavior [59], [66], but no differences were found between any of the experimental groups and these data are not further discussed.

### Elevated Plus-Maze

For analysis of anxiety-related behavior, resident females were tested on the elevated plus-maze (EPM) in the early dark phase (13.00–16.00 h) with lights on. The EPM consists of a plus-shaped platform elevated 80 cm above the floor, with two open (50×10 cm; 100 lux) and two closed arms (50×10×40 cm; 20 lux). Rats were placed in the center square facing a closed arm. The following parameters were recorded during the 5-min test using a video/computer system (Plus-maze version 2.0; Ernst Fricke): time spent in open and closed arms, number of entries into open and closed arms, latency to enter an open arm. Here, statistical analysis is only presented for the percentage of time spent on the open arms: [time on open arms]/[time on open+closed arms] × 100% as indication of anxiety levels.

### Determination of Estrous Phase

In experiment 1, the estrous phases of residents and intruders were determined using vaginal smears that were taken with moistened cotton swabs, spread on object glasses, dipped in Nissl solution, and dehydrated in an alcohol series. The predominant presence of nucleated epithelial cells (proestrus), anucleated cornified epithelial cells (estrus), leukocytes (metestrus), or a low number of mixed cells (diestrus) in the smears was assessed under a microscope [67]. Residents and intruders were subsequently pooled into two categories: receptive (proestrus/estrus) or non-receptive (metestrus/diestrus). Vaginal smears were taken after the FIT, to avoid stress-induced alterations in aggressive behavior [61].

### Cannula Placement

For stereotaxic implantation of ICV guide cannulae, rats were anesthetized (isoflurane, Forene®, Abbott GmbH & Co. KG, Wiesbaden, Germany), injected i.p. with an antibiotic (3.0 mg/120 µl/animal Baytril®, Bayer Vital GmbH, Leverkusen, Germany), and mounted on a stereotaxic frame. An ICV guide cannula (21 G, 12 mm; Injecta GmbH, Germany) was implanted resting 2 mm above the lateral ventricle (1.0 mm caudal to bregma, 1.6 mm lateral to midline, 2.0 mm beneath the skull surface, [68]), fixed to the skull with two jeweller’s screws and dental cement (Kallocryl, Speiko-Dr. Speier GmbH, Muenster, Germany), and closed by a stainless steel stylet (25 G). After surgery, each rat was allowed to recover for 48 h in her observation cage; stylets were cleaned on the day between surgery and the FIT.

### ICV Infusion

Synthetic OXT (Sigma Aldrich Biochemicals, Steinheim, Germany) was infused at a dose of 0.1 µg/5 µl. This dose was carefully chosen to be low enough to avoid stereotyped barrel rotations (as often seen in female Wistar rats at 1.0 µg/5 µl, personal communication, M. Lukas), but high enough to reliably affect social behaviors in male rats [50]). For ICV infusion of either synthetic OXT or VEH (5 µl Ringer’s solution, pH = 7.4, B. Braun AG, Melsungen, Germany), a 25 G infusion cannula extending 2 mm beyond the guide cannula and connected via polyethylene tubing to a Hamilton syringe was inserted into the guide cannula. After slow manual infusion the system was left in place for 10 s. Infusions took place 20 min prior to the FIT. After completion of the experiment, cannula placement was verified post mortem by ICV infusion of 2–3 µl black ink (Pelikan 4001, Hannover, Germany).

### Immunohistochemistry

For immunohistochemical staining of pERK and OXT, rats were deeply anesthetized with isoflurane (Forane, Baxter, Deerfield, IL, USA) immediately after the end of the 10-min FIT and perfused transcardially with 0.1 M phosphate-buffered saline (PBS) followed by 4% paraformaldehyde (PFA) in an adjacent room. All 4% PFA perfusions started within 3 min after the end of the FIT. Fixated brains were dissected and immersed in 40 ml 4% PFA and post-fixated for 1 h before being stored in 0.1 M PBS at 4°C. Three to four days prior to cutting, brains were submerged in 30% phosphate buffered sucrose until saturated. The cryoprotected brains were snap-frozen on dry ice and cut into 40-µm thick sections on a cryostat set at −19°C. Consecutive sections were collected in six vials containing 5 ml 0.1 M PBS, to enable up to six different immunohistochemical stainings. Brain tissue was protected against mold with 50 µl 0.01% sodium azide per vial.

For single pERK staining, one series of brain sections (from a total of six) per animal was placed in a well on a 24-wells plate containing 3 ml of 0.1 M PBS. Brain sections from all individuals in the experiment were stained simultaneously. Staining started by washing the sections 3×10 min with fresh 0.1 M PBS, followed by 30 min of 0.1% H_2_O_2_ and another round of 3×10 min 0.1 M PBS. Tissue was then pre-incubated for 30 min with 0.1 M PBS containing 0.1% bovine serum albumin and 0.3% Triton-X-100 (PBS-BT) and incubated overnight at 4°C with PBS-BT containing rabbit-anti-pERK antibody (1∶250, #4370, Cell Signaling, Danvers, MA, USA). The next day, tissue was washed 3×20 min with 0.1 M PBS. Brains were then incubated for 90 min with PBS-BT containing biotinylated goat-anti-rabbit antibody (1∶200, BA-1000, Vector Laboratories, Inc., Burlingame, CA, USA), followed by 3×20 min washing with 0.1 M PBS. Staining was enhanced by 90 min of incubation with PBS-BT containing ABC-vector (1∶800, PK-6100, Vector Laboratories, Inc., Burlingame, CA, USA), followed by 3×20 min washing with 0.1 M PBS. Tissue was then stained with a DAB-staining kit (SK-4100, Vector Laboratories, Inc., Burlingame, CA, USA) resulting in a blue-black cytoplasmic staining, and washed 3×15 min in 0.1 M PBS. Brain sections were mounted on adhesive microscope slides (Superfrost® Plus, Thermo Fisher Scientific Inc., Waltham, MA, USA), dehydrated in increasing ethanol concentrations, cleared and embedded in Roti®-Histol and Roti®-Histokitt (Carl Roth GmbH, Karslruhe, Germany), and coverslipped.

For pERK and OXT double staining, four brain sections containing the PVN were selected for each individual, and placed in a well (one per individual) on a 24-wells plate containing 1 ml of 0.1 M PBS. Again, brain sections from all individuals in the experiment were stained simultaneously. Staining started by washing the sections 3×10 min with fresh 0.1 M PBS, followed by 10 min incubation in ice-cold methanol at −20°C, 1 h of incubation in blocking solution (0.1 M PBS with 5% normal goat serum and 0.3% triton-x-100), and 64 h of incubation in antibody solution (0.1 M PBS with 1% bovine serum albumin and 0.3% triton-x-100) containing rabbit-anti-pERK antibody (1∶250, Cell Signaling #4370, Danvers, MA, USA) and mouse-anti-OXT antibody (1∶400, kind gift from Prof. Dr. Gainer, NIH, Bethesda, USA, 60). After incubation, brain sections were washed 3×10 min in 0.1 M PBS and (in the dark) incubated for 120 min with green fluorescent goat-anti-rabbit antibody (Dy-Light®488, 1∶200, DI-1488, Vector Laboratories, Inc., Burlingame, CA, USA) followed by 3×10 min washing in 0.1 M PBS and 120 min incubation in red fluorescent goat-anti-mouse antibody (Dy-Light®594, 1∶200, DI-2549, Vector Laboratories, Inc., Burlingame, CA, USA). Finally, brain sections were washed 3×10 min in 0.1 M PBS, mounted on adhesive microscope slides (Superfrost® Plus, Thermo Fisher Scientific Inc., Waltham, MA, USA) and embedded in Vectastain hard-set mounting medium (H-1400, Vector Laboratories, Inc., Burlingame, CA, USA). Slides were kept overnight in the dark at 4°C.

### Immunohistochemical Analysis

Single labeling of pERK was analyzed in ten brain areas as derived from the Paxinos rat brain atlas [68]: the ventral part of the lateral septal nucleus (LSv), the medial division of the posteromedial bed nucleus of the stria terminalis (BSTmpm), the medial parvocellular and lateral magnocellular parts of the paraventricular hypothalamic nucleus (PVNmp and PVNlm), the hypothalamic attack area (HAA), the ventrolateral part of the ventromedial hypothalamic nucleus (VMHvl), the posterodorsal medial and central parts of the amydaloid nucleus (MeApd, CeA), the dorsal part of the dorsal raphe nucleus (DRd) and the ventrolateral periaqueductal gray (PAGvl). These brain areas were selected based on two criteria: a known role in aggressive behavior [15], [27]–[31], and a considerable presence of pERK-IR in at least one of the two experimental groups. For each brain area, the optimal section was selected using a Leica DM5000B microscope connected to a personal computer; a photograph was taken at 10x magnification using Leica Application Suite v3.7 software. The number of pERK-IR neurons was counted in a 200×200 µm square placed over a representative area within the selected nucleus, using ImageJ software (NIH).

Double labeling of pERK and OXT was analyzed using the same microscope. For each individual the section with the highest density of OXT neurons in the PVNlm was selected. Images (one per animal, either left or right half of the selected PVNlm) were taken from the red and green channels without adjusting the focal plane in between, using L400W software installed on a personal computer connected to the microscope. Images were imported into Fiji (http://fiji.sc/wiki/index.php/Fiji), which is an open source image-processing package based on ImageJ software (National Institutes of Health). In each image the background was subtracted and the brightness and contrast adjusted so that cell bodies were highlighted and axons de-emphasized. Then, the two-channel readings for green and red fluorescence were merged and an area of 800×800 pixels containing the highest density of OXT neurons was defined. Within this area, single (OXT, pERK) and double (OXT+pERK) labeled neurons were counted; only neurons that demonstrated the same morphology, orientation and position in both red and green were considered doubly labeled.

### Statistics

All data were analyzed by SPSS 19.0 software. As the assumption of normality was strongly violated for our most important behavioral parameters (i.e., skewed to the right for attack frequency and percentage of time spent behaving aggressively, skewed to the left for attack latency; Shapiro-Wilk tests: p<0.01), behavioral data are presented as medians and quartiles and non-parametric tests were used to analyze behavior. Overall differences in behavior between multiple experimental groups were analyzed using Kruskal-Wallis tests, followed (when appropriate) by post-hoc pair-wise comparisons using Mann-Whitney tests for between-subjects analyses or Wilcoxon rank tests for within-subjects analyses (i.e., changes in behavior over time). Fisher’s exact test (4×2 design) was used to analyze the proportion of females attacking in each of the four estrous cycle groups. Correlations between anxiety behavior in the EPM and aggressive behavior in the FIT were analyzed using the non-parametric Spearman’s correlation coefficient (ρ). Neuronal activation in tolerant and aggressive females was compared using student’s t-tests. All tests were performed two-sided and the level of significance was set at p<0.05, but strong trends (p<0.07) are reported and discussed as well.

## Results

### Exp. 1. Overall Behavior in the FIT: No Effect of Estrous Cycle (Fig. 2, Table 1)

**Figure 2:**
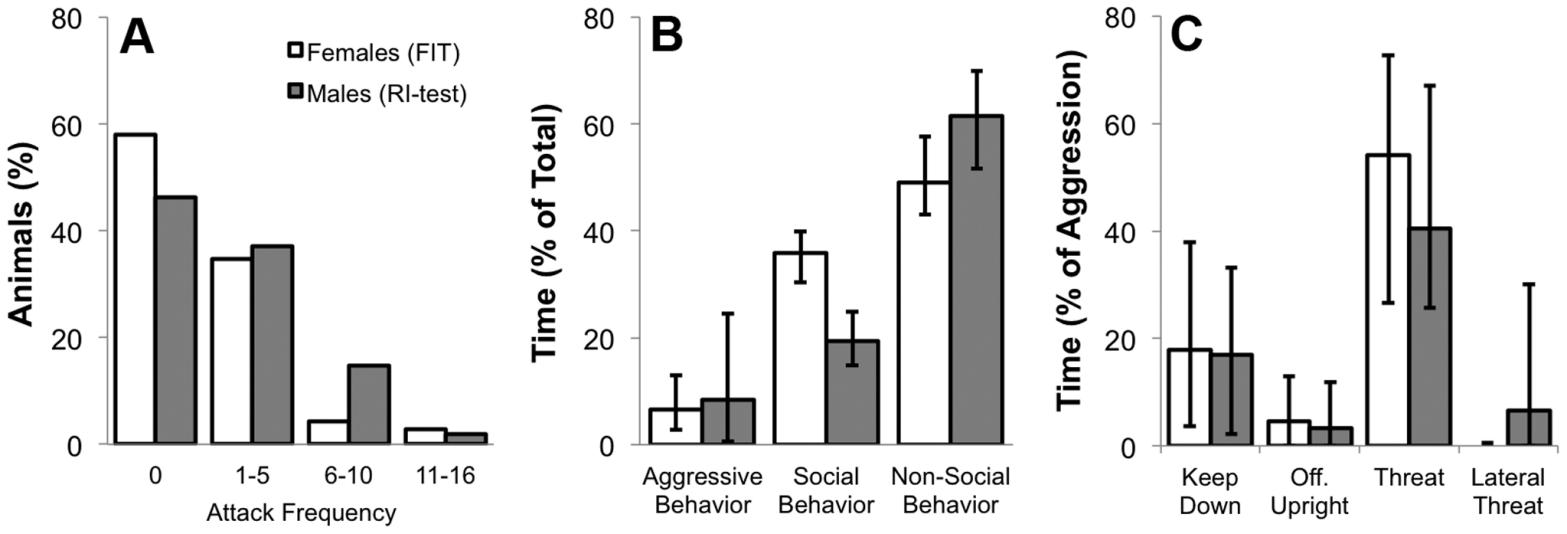
Behavioral profile of adult (10–11 week old) virgin female NAB residents (n = 69) during their first 10-min female intruder test (FIT), and adult (16–22 week old) male NAB residents (n = 108) during their first 10-min resident-intruder test (RI-test). **A:** attack frequency distribution; **B:** aggressive, social and non-social behavior as percentage of total behavior; **C:** different types of offensive behavior as percentage of total aggressive behavior. Data are medians and quartiles. A statistical comparison of the sexes was not performed.

The behavior of young female NAB rats in the FIT (n = 69) was analyzed and compared with the behavior of 108 adult male NAB rats in the RI-test. Due to the difference in age and housing conditions between females tested in the FIT and males tested in the RI-test, a statistical analysis was deemed inappropriate. The following comparison serves merely an illustrative purpose. Approximately 40% of the females (compared to 54% of males) attacked the intruder, with a marked variability in attack frequency (1 to 16 attacks for both females and males, Fig. 2A). Female residents spent 6.7% of the time displaying aggressive behavior (compared to 8.3% in males, Fig. 2B), and showed the same types of offensive behavior as male residents (Fig. 2C). Both female and male residents spent the largest proportion of the 10-min test displaying non-social behaviors (∼50–60%; exploration, auto-grooming, eating/drinking, Fig. 2B), but females spent more time displaying non-aggressive social behavior, i.e. investigating the intruder, compared with males (∼35% vs. 20%, Fig. 2B). Defensive and sexual behaviors were only occasionally observed in female rats (data not shown). Percentage of time spent behaving aggressively did not correlate with the difference in body weight between resident and intruder (ρ = −0.132, p = 0.278).

The four estrous cycle groups (receptive residents confronted with either receptive or non-receptive intruders; non-receptive residents confronted with either receptive or non-receptive intruders) did not differ in the proportion of residents that attacked the intruder (Fisher’s exact test: P = 0.43), nor in percentage of aggressive, social and non-social behaviors displayed in the FIT (for statistical analysis, see Table 1).

**Table 1:** Effect of estrous phase on behavior in the FIT.

Phase Resident:	Receptive		Non-Receptive		χ^2^(3); P
Phase Intruder:	Rec.	Non-Rec.	Rec.	Non-Rec.	
N (total = 69):	15	20	11	23	
Fem. Attacking	5 (33%)	11 (55%)	3 (27%)	10 (43%)	*[Fisher: P = 0.43]*
Aggressive Behavior	8.90; 3.9−12.5	9.24; 5.1−12.5	3.82; 2.2−6.42	6.19; 0.7–18.0	4.13; 0.25
Social Behavior	35.32; 30.6−39.1	32.72; 28.8−41.4	38.83; 36.6−41.5	34.72; 31.7−38.1	3.73; 0.29
Non-Social Behavior	51.60; 43.0−60.0	46.39; 41.4−55.0	49.76; 48.3−55.5	48.79; 45.0−57.5	1.33; 0.72

The number of females attacking as well as the percentage aggressive, social and non-social behavior of receptive and non-receptive adult (10–11 week old) virgin female NAB residents in response to receptive and non-receptive virgin female intruder rats in a 10-min Female Intruder Test (FIT). Data are medians and quartiles.

### Exp. 2. Effects of Innate Anxiety on Female Aggressive Behavior (Fig. 3, Table 2)

**Figure 3:**
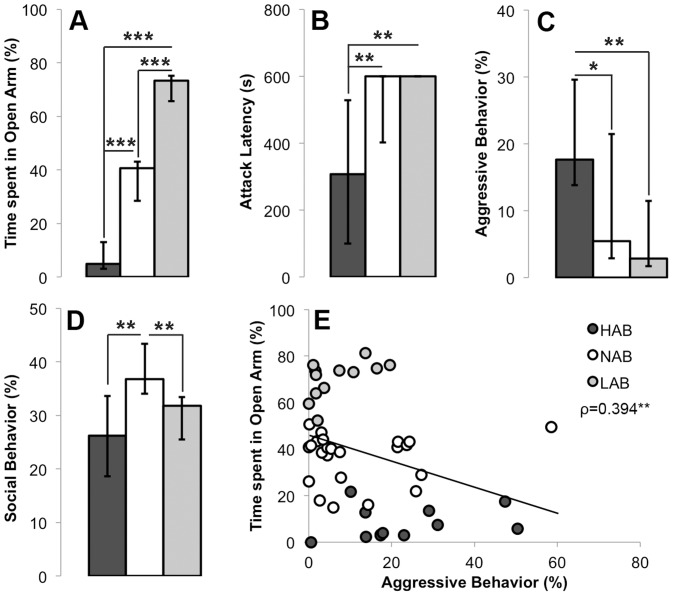
Behavioral profile of adolescent (7–8 week old) virgin female HAB (n = 12), NAB (n = 23) and LAB (n = 12) rats in the EPM and FIT. **A:** Percentage of time spent in the open arms of the EPM; **B–D:** Attack latency, percentage of time displaying aggressive behavior and percentage of time displaying social behavior in a 10-min FIT. Data in **A–D** are medians and quartiles. **E:** Spearman’s correlation coefficient between percentage of time spent in the open arms of the EPM and percentage of time spent displaying aggressive behavior in the FIT two days later. *P<0.05; **P<0.01; ***P<0.001.

Adolescent (and adult, data not shown) HAB, NAB and LAB females differed significantly in the percentage of time spent on the open arms of the EPM (Fig. 3A). As expected, HAB females spent the least time in the open arms and LAB females the most. In the FIT, adolescent HAB, NAB and LAB rats differed significantly in attack latency (Fig. 3B) and attack frequency (data not shown), as well as percentage of time displaying aggressive behavior (Fig. 3C), social behavior (Fig. 3D) and non-social behavior (data not shown). Adolescent HAB females had shorter attack latencies, higher attack frequencies and spent more time displaying aggressive behavior than NAB and LAB females. In contrast, adult HAB, NAB and LAB rats did not differ in any aspect of aggression (see exp. 3). In addition, both adolescent and adult NAB females spent more time displaying non-aggressive social behavior than HAB and LAB females (for adolescents: see Table 2; for adults: χ^2^(2) = 13.33, p = 0.001; NAB vs LAB and HAB: Z<−2.73, p<0.01). Lastly, adolescent but not adult LAB females spent more time displaying non-social behaviors compared to HAB and NAB females. For statistical analysis, see Table 2.

**Table 2:** Effect of innate anxiety levels on behavior in the FIT.

Behavior	Test	HAB/NAB/LAB χ^2^(2); P	Pair-wise Comparison	Z
Time in Open Arm (%)	EPM	38.18; <0.001	HAB vs. NABHAB vs. LABNAB vs. LAB	−4.62***−4.18***4.80***
Attack Latency (s)	FIT	11.82; <0.01	HAB vs. NABHAB vs. LABNAB vs. LAB	−2.78**−2.96**−−−
Attack Frequency (n)	FIT	13.52; <0.01	HAB vs. NABHAB vs. LABNAB vs. LAB	−2.91**−3.21**−−−
Social Behavior (%)	FIT	13.83; <0.01	HAB vs. NABHAB vs. LABNAB vs. LAB	−3.27**−2.64**−−−
Aggressive Behavior (%)	FIT	10.32; <0.01	HAB vs. NABHAB vs. LABNAB vs. LAB	−2.26*−3.41**−−−
Non-social Behavior (%)	FIT	6.58; <0.05	HAB vs. NABHAB vs. LABNAB vs. LAB	−−−−2.31*−2.22*

Overall effects (Kruskal-Wallis) and Mann-Whitney pair-wise comparisons for experiment 2 (Fig. 3).

***P<0.001,

**P<0.01,

*P<0.05.

The level of anxiety correlated positively with the level of aggression (i.e., the percentage of time spent on the open arm of the EPM correlated *negatively* with the percentage of time spent behaving aggressively in the FIT) when adolescent females from all three selection lines were pooled (ρ = 0.394, p = 0.006, fig. 3E). However, no significant correlations between anxiety and aggression were found within the three selection lines (HAB: ρ = 0.301, NAB: ρ = −0.072, LAB: ρ = 0.126; p>0.342).

### Exp. 3. ICV OXT Inhibits Aggressive Behavior in Adult NAB and LAB Females (Fig. 4, Table 3)

Adult LAB and NAB females were found to be more aggressive in FIT 2 compared with FIT 1 when treated with VEH prior to FIT 2, whereas HAB rats showed no change after repeated testing. This increase in aggression seen in LAB and NAB rats was prevented by acute ICV OXT infusion prior to FIT 2. As a result, OXT-treated NAB and LAB females displayed significantly less aggressive behavior (percentage of time) compared with respective VEH groups. Infusion of OXT did not alter the percentage of social behavior compared with VEH in any of the rat lines. For statistical analysis see Table 3.

**Table 3:** Effect of ICV infusion of OXT on behavior in the FIT.

Comparison:		OXT vs VEH	FIT 2 vs FIT 1	
Behavior	Rat Line	Z	Z [VEH]	Z [OXT]
Aggressive Behavior (%)	HABNABLAB	−−−−−3.02**−2.95**	−−−−−1.89#−2.60**	−−−−−−−−−−−−
Attack Latency (s)	HABNABLAB	−−−−−−−−−−−−	−−−−−−−−−2.29*	−−−−−−−−−−−−
Social Behavior (%)	HABNABLAB	−−−−−−−−−−−−	−−−−−−−−−2.10*	−−−−−−−−−−−−
Non-social Behavior (%)	HABNABLAB	−−−−−1.89#−2.04*	−−−−−−−−−−−−	−−−−−−−−−−−−

**Figure 4:**
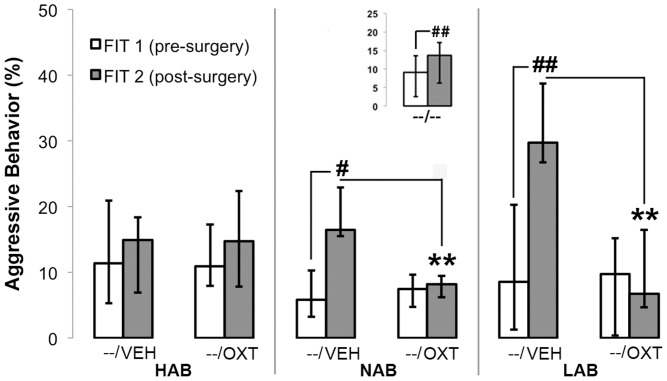
Percentage of aggressive behavior of adult (10–11 week old) virgin female HAB, NAB and LAB rats in two 10-min FITs, five days prior to surgery (FIT 1) or two days after surgery (FIT 2). 20(5 µl Ringer, n = 10 per selection line) or OXT (0.1 µg/5 µl Ringer, n = 10 per selection line). Insert shows data for unmanipulated NAB rats (no surgery performed, n = 24). **Significant difference with corresponding VEH data (p<0.01). ^#/##^Significant difference with corresponding FIT 1 data (^#^p = 0.06, ^##^P<0.05).

Mann-Whitney pair-wise comparisons for experiment 3 (Fig. 4).

**P<0.01,

*P<0.05;

# P = 0.06.

As a reference, 24 adult NAB females (10–11 weeks of age) were tested in the FIT twice, without surgical or any other intervention. 18 out of 24 females showed an increased percentage of aggressive behavior in FIT 2 compared with FIT 1 (Wilcoxon test for paired samples: Z = −2.114, p = 0.034; insert Fig. 4).

### Exp. 4. Aggressive Behavior is Associated with Altered pERK-IR (Fig. 5 and 6)

Behavioral analysis of 23 adolescent NAB females resulted in two experimental groups (n = 7 each, 9 females were excluded for reasons explained above) that differed significantly in the median percentage of aggressive behavior (non-aggressive females: 4.5% (quartiles: 3.8–5.7%); aggressive females: 24.3% (22.5–26.6%); Z = −3.13, p<0.01) and percentage of non-social behaviors (non-aggressive females: 45.2% (44.9–51.2%); aggressive females: 39.8% (30.1–41.2%), Z = −2.62, p<0.01). Both groups showed a similar amount of non-aggressive social behaviors (Z = −1.09, p = 0.28).

**Figure 5:**
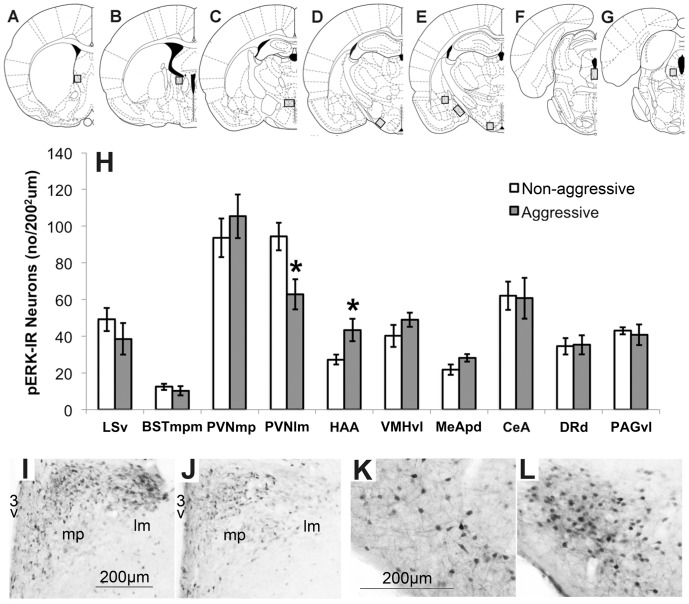
Immunohistochemical single-labeling of pERK. **A–G:** schematic drawings of coronal brain sections (derived from the Paxinos rat brain atlas, 63) with grey squares corresponding to the location of the quantified brain areas (**A:** LSv, bregma +0.20; **B:** BSTmpm**,** bregma −0.80; **C**: PVNm and PVNl, bregma −1.80; **D:** HAA, bregma −2.80; **E:** MeApd, CeA, VHMvl**,** bregma −3.14; **F:** DRd, bregma −7.80; **G:** PAGvl, bregma −8.30); **H:** Average density of pERK-IR in non-aggressive (white bars, n = 7) compared with aggressive (grey bars, n = 7) adolescent (7–8 week old) female NAB rats. Data are means ± s.e.m; *P<0.05; **I–J:** Illustration of pERK-IR in the PVNmp (“mp”) and PVNlm (“lm”) of non-aggressive (left) and aggressive (right) individuals (3v = third ventricle); **K–L:** Illustration of pERK-IR in the HAA of non-aggressive (left) and aggressive (right) individuals.

Females that had been aggressive in the FIT showed a lower level of pERK-IR in the PVNml (t = −2.81, p<0.05) and increased pERK-IR in the HAA (t = 2.41, p<0.05) compared with non-aggressive females (Fig. 5). Interestingly, receptive residents showed reduced pERK-IR in the BSTmpm compared to non-receptive residents (14.6±1.9 vs. 8.1±1.6, p<0.05, data not shown), independent of the amount of aggressive behavior.

**Figure 6:**
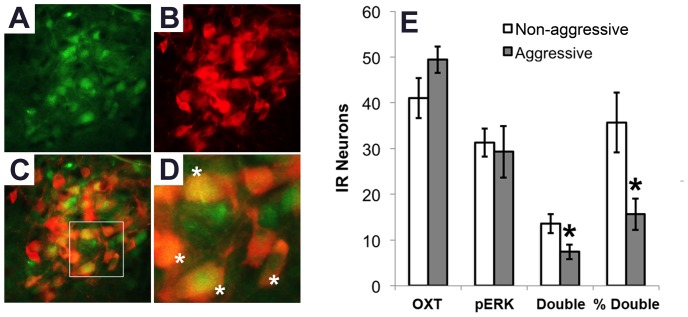
Immunohistochemical double-labeling of OXT and pERK. **A–C:** Illustrations of OXT (green) and pERK (red) IF in the PVNlm of a non-aggressive female. Total area of A, B and C corresponds to the size of the analyzed area; **D:** Higher magnification of the white square in C, white asterisks signify double-labeled neurons; **E:** Density of single- and double-labeled neurons as well as the percentage of OXT-IR neurons that also showed pERK-IR in non-aggressive (white bars, n = 7) and aggressive (grey bars, n = 7) adolescent female NAB rats. Data are means ± s.e.m; *P<0.05.

Double labeling immunofluorescence for OXT and pERK in the PVNlm (Fig. 6) revealed that aggressive females showed a reduced number of double-labeled neurons (t = −2.37, p<0.05) and, likewise, a reduced percentage of OXT neurons that showed pERK-IR (t = −2.72, p<0.05).

## Discussion

The experiments reported here confirm that aggression is an important component of the behavioral repertoire of adolescent and adult virgin female rats, and can be reliably quantified in the FIT, i.e. during confrontation with a smaller female intruder rat following 48 h of isolation. Further, female aggression seems to be independent of the estrous cycle of both residents and intruders, but is correlated with the innate level of anxiety-related behavior with highest aggression levels found in adolescent HAB females. Female aggression is associated with reduced neuronal activity in the hypothalamic PVN, specifically in local OXT neurons, and central infusion of OXT results in reduced aggression in the FIT. These results indicate an important involvement of the brain OXT system in female aggression regulation.

We compared behavior of female residents in the FIT with behavior of male residents in the RI-test (based on recent results, see [63]). Although the differences between the paradigms (housing conditions, age of testing) preclude a direct statistical comparison of the sexes, a descriptive comparison of the behavior of male and female residents during their first exposure to the FIT or RI-test can be made. Overall, female residents showed a somewhat lower level of aggression compared with males (42% vs. 54% attacked their intruder at least once, median time spent displaying aggression was 6.7% vs. 8.3%). These differences between the sexes were much smaller than expected, however, a direct comparison of males and females of the same age in the same paradigm is needed to draw any definite conclusions. Nevertheless, combined with the recent report of considerable aggression in female Wistar rats in a different paradigm [47], these data point in the direction of a persistent under-estimation in the literature of female aggression in rats.

It should be noted here that the 48 h isolation period in females was, in contrast to the 10 days of pair-housing in males, not intended to instigate aggression, but rather to allow recovery from stereotactic surgeries needed for (for example) neuropharmacological intervention. In order to enable comparisons between various experiments, we standardized the isolation period at least in this set of studies. However, significantly shorter or longer bouts of isolation, as well as socially instable housing paradigms [69], are expected to alter general social and aggressive interactions, which would need to be studied in more detail.

Our finding of a similar median percentage of time being aggressive of non-receptive and receptive residents, independent of the estrous cycle of the intruder, is consistent with results reported by Cordero et al. [47]. However, Ho et al. [46] reported reduced aggression in receptive adult female Wistar rats. The difference in findings can be explained by the experimental conditions in the latter study, in which the females were sexually experienced, had been pseudo-pregnant, were isolated for at least four weeks prior to the experiment and were confronted with same-size, ovariectomized (i.e., non-receptive) intruder females. These experiences are likely to have significantly altered their behavior toward an intruder. Nevertheless, determining the estrous phase of the residents and intruders following the FIT remains advisable to confirm our findings under different experimental conditions or in other rat strains.

High levels of innate anxiety were associated with increased aggression in adolescent females, but this association disappeared in adulthood. In adolescence as well as in adulthood, HAB (and LAB) females displayed less social behavior toward their intruder than NAB females. These findings are consistent with other results from our lab showing altered social behavior in HAB females. For example, 5-week old female HAB juveniles display significantly reduced play-fighting behavior accompanied by reduced ultrasonic vocalizations compared with NAB and LAB juveniles (M. Lukas, personal communication). In addition, lactating HAB females show increased levels of maternal aggression compared to LAB and NAB dams [35]. In humans, anxiety disorders (especially social phobia) are often co-morbid with behavioral problems including aggression, especially during adolescence [70], [71], and this link appears to be even stronger in girls compared to boys [72], [73]. Taken together, juvenile and adolescent HAB females may form a promising animal model to further explore the link between high anxiety and aggression.

ICV infusion of synthetic OXT inhibited aggression in both NAB and LAB females. The demonstration of an anti-aggressive effect of OXT in virgin female rats is in agreement with recent findings in adult male wild-type Groningen rats [59]. However, our effects were found at a 10-fold lower dose (0.1 versus 1 µg/5 µl), potentially because female rats are more sensitive to the anti-aggressive effects of OXT. Our findings are also in concordance with the results of Harmon et al. [58], who showed inhibition of aggression by OXT in virgin female Syrian hamsters. The capacity of central OXT to reduce aggression in virgin females has to be clearly separated from its substantial promotion of maternal aggression in lactating animals. Maternal aggression is part of the complex patterns of maternal behavior to protect the offspring [42], and OXT was shown to facilitate the defensive behavior of lactating dams specifically within the PVN [35] and the amygdala [74]. Future research is needed to further determine the extent to which the neuronal circuitries involved in female aggression of virgin rats and of lactating dams differ.

In the present experiment, HAB females did not reduce their aggressive behavior in response to OXT infusion, in contrast to NAB and LAB females. This suggests a difference in the neurobiological underpinnings of aggressive behavior in the different rat lines. Indeed, HAB rats are known to overexpress AVP due to impaired repression at a AVP promotor polymorphism [75]. Increased AVP exerts a strong control over aggression in males [18], [76], and lactating HAB females show increased release of AVP in the central amygdala during maternal defense [77]. Future research is needed to confirm a role of AVP in aggressive adolescent female HAB rats.

Vehicle-treated LAB rats and, to a lesser extent, NAB rats showed increased aggression when exposed to the FIT a second time. Surgery and subsequent vehicle-infusion resulting in increased levels of stress and anxiety may have contributed to the increased aggression. However, a similar increase was seen in non-manipulated NAB females tested twice in the FIT without surgery in between. These findings are consistent with multiple reports of increased aggression induced by fighting experience in male rats and house mice, and in male and female California mice and Syrian hamsters [61], [78]–[82].

To this date virtually nothing is known about the neurobiological underpinnings of non-maternal female aggressive behavior, especially in the gregarious rat. Therefore, we analyzed local patterns of neuronal activation (density of pERK-IR neurons) in aggressive and non-aggressive female residents [61], [62]. Interestingly, aggressive females had a lower number of pERK-IR neurons in the PVNlm compared to non-aggressive females. Since this difference in pERK-IR was found at the approximate location of OXT neurons, double labeling immunofluorescence was performed confirming that aggressive females had reduced pERK-IR in OXT neurons compared to tolerant females. This finding is consistent with results from female California mice housed under shortened day lengths that showed both increased aggressive behavior and reduced immunoreactivity of pERK and oxytocin in the PVN, as well as reduced OXT release in the blood following an RI-test [62]. Although these results do not demonstrate a causal link, they imply that the increased level of pERK-IR in OXT neurons in non-aggressive females is related to increased OXT release within the PVN and/or central target regions, the more as the ERK1/2 pathway is functionally able to alter the firing of OXT neurons [83]. Combined with the anti-aggressive actions of central OXT infusion in female rats, we postulate that the level of aggressive behavior toward an intruder displayed by a female rat depends at least in part on the activity of the brain OXT system and the central release of OXT. A similar pathway may be present in male rats [59], [84].

Aggressive females showed a higher density of pERK-IR neurons in the HAA, which is consistent with the increased neuronal activation in this area in aggressive (male or lactating female) rats and mice [30], [37], [85], [86] as well as with the well-known pro-aggressive function of this brain area demonstrated in male rodents [87]. Substantial pERK-IR was found in LSv, BSTmpm, PVNmp, VMHvl, CeA, MeApd, DRd and PAGvl in FIT-exposed females, but no differences existed between non-aggressive and aggressive individuals. Further research is needed to study the neurobiological pathways and neuronal activation underlying female aggression in more detail, for example using different markers of neuronal activation such as Fos or Egr-1 (zif268).

In conclusion, aggressive behavior in virgin female Wistar rats can be monitored using the FIT – a simple modification of the RI-test established for studying inter-male aggression. Our findings show a strong inhibiting effect of OXT on female aggression, and the association between low brain OXT system activity and high female aggression. Future studies are needed to localize the effects of OXT, to show the involvement of the endogenous OXT system, and to investigate the moderating roles of AVP, 5-HT and steroid hormones (which are known to influence inter-male aggression) on female aggression. Together, these data will shed light on the, putatively sex-specific, neurobiological control of aggressive behavior in young girls and women.
